# Comparative analysis of two *Porphyridium* species for phycobiliprotein and polysaccharide production under different photoperiods

**DOI:** 10.1186/s40643-025-00996-0

**Published:** 2025-12-26

**Authors:** Liang Ji, Luxuan Xu, Zhangzhen Chen, Yulong He, Artem Yurevich Manyakhin, Pengfei Cheng, Liyun Sun, Jianhua Fan

**Affiliations:** 1 State Key Laboratory of Bioreactor Engineering, East China University of Science and Technology, 130 Meilong Road, Shanghai, 200237 People’s Republic of China; 2 Department of Applied Biology, East China University of Science and Technology, 130 Meilong Road, Shanghai, 200237 People’s Republic of China; 3 School of Chemistry and Chemical EngineeringState Key Laboratory Incubation Base for GreenProcessing of Chemical Engineering, Shihezi University, Shihezi, 832003 People’s Republic of China; 4 Far Eastern Branch, Federal Scientific Center of the East Asia Terrestrial Biodiversity, Russian Academy of Sciences, Vladivostok, Russia 690022; 5 College of Food Science and Engineering, Ningbo University, Ningbo, 315211 Zhejiang People’s Republic of China

**Keywords:** *Porphyridium purpureum*, *Porphyridium aerugineum*, Phycoerythrin, Phycocyanin, Polysaccharide

## Abstract

Phycobiliproteins and microalgal exopolysaccharides serve as natural pigments and functional additives in food applications. Current production primarily relies on multicellular algae, where yields are constrained by challenges in achieving high-density cultivation. This study investigated photoperiod effects on growth and production of phycobiliproteins and polysaccharides in two unicellular red algae (*Porphyridium purpureum* and *Porphyridium aerugineum*). Results demonstrated that short photoperiods enhanced the accumulation of core phycobiliproteins, specifically by increasing phycoerythrin in *P. purpureum* to a maximum of 30.5 ± 0.8 mg/g DW and phycocyanin in *P. aerugineum* to 41.9 ± 0.2 mg/g DW. Conversely, long photoperiods promoted biomass accumulation, yielding peak phycoerythrin production (140.6 ± 0.9 mg/L) in *P. purpureum* and phycocyanin (137.7 ± 1.2 mg/L) in *P. aerugineum* at day 12. Both species exhibited superior exopolysaccharide production under long photoperiods, though *P. purpureum* showed significantly higher productivity (898.7 ± 41.0 mg/L at day 20). These findings offer strategic solutions for sustainable production of food-grade pigments and polysaccharides through optimized unicellular algal cultivation.

**Graphical abstract:**

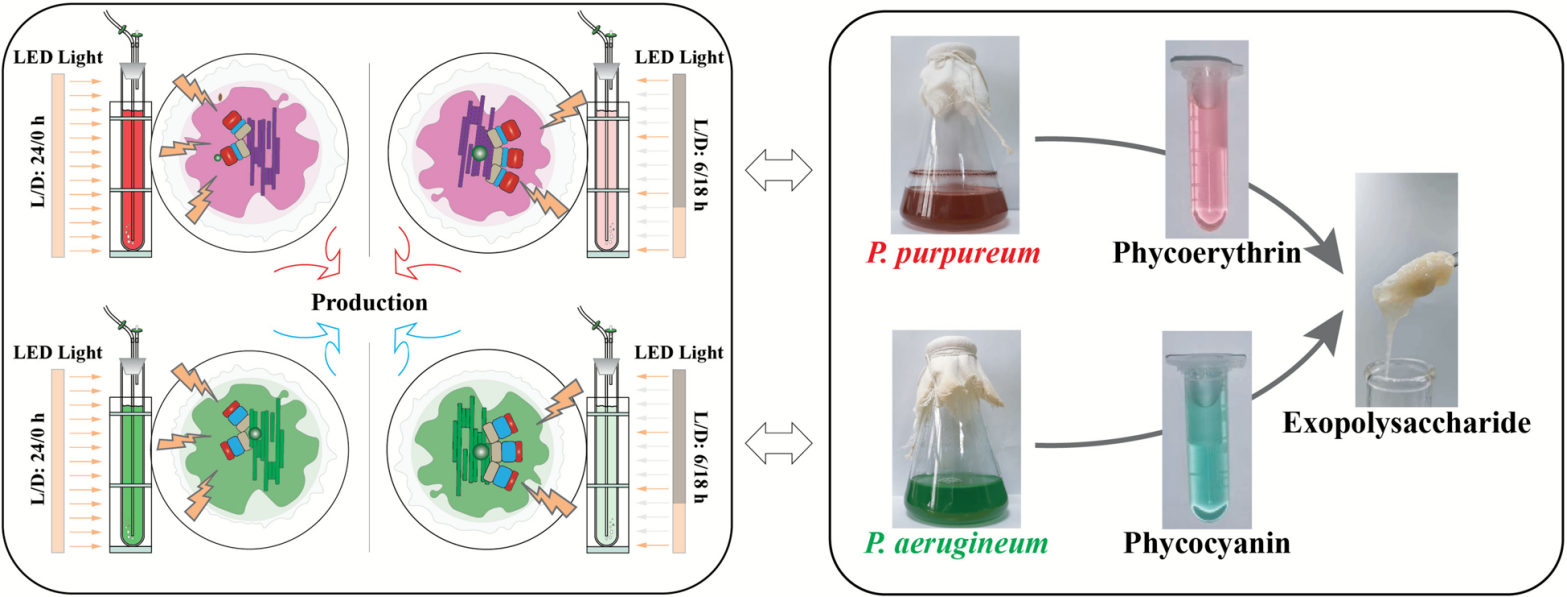

## Introduction

As the global population approaches a projected 10 billion by 2050, arable land resources face intensifying constraints from degradation, scarcity, and urban expansion (Dai et al. 2025). Concurrently, climate change threatens agricultural productivity through increased extreme weather events, collectively heightening future global food shortage risks (Abebaw 2025). This urgency necessitates novel, sustainable food sources. Microalgae emerge as a highly promising solution, offering an exceptional nutritional profile that includes high-quality proteins, lipids containing unsaturated fatty acids, vitamins, and bioactive compounds (Sarıtaş et al. 2024). Crucially, their cultivation requires no arable land, utilizing non-arable land or closed systems instead, while exhibiting highly efficient photosynthetic carbon fixation (Chen et al. 2022). These properties enable microalgae to provide nutrient-dense food while simultaneously mitigating greenhouse gas emissions, positioning them as a key potential resource for addressing the dual challenges of food security and sustainable development (Ahmad et al. 2022).

Phycobiliproteins, primarily found in red algae (rhodophytes) and cyanobacteria, serve as light-harvesting antenna complexes and exhibit distinctive fluorescent properties that enhance their value in food applications (Ji et al. 2023). Structurally classified into phycoerythrin (PE), phycocyanin (PC), and allophycocyanin (APC), PE and PC offer vibrant colors and excellent water solubility (Kumar et al. 2025). This makes them ideal natural replacements for synthetic food colorants in beverages, dairy products, and confectionery, effectively avoiding allergenicity and toxicity concerns (Galetovic et al. 2020; Vieira et al. 2025). Beyond providing pigmentation, phycobiliproteins act as bioactive additives. Research confirms their significant antioxidant activity, which extends food shelf-life (Ahmadi et al. 2024). Additionally, their anti-inflammatory and immunomodulatory capabilities support their use in specialized functional foods designed for immune enhancement (Mysliwa-Kurdziel and Solymosi 2017).

Microalgal exopolysaccharides (EPS) are natural high-molecular-weight carbohydrates secreted during microalgal growth, characterized by complex structures of glucose, galactose, or mannose monomers linked through glycosidic bonds (He et al. 2025). These structures often incorporate sulfate groups that critically define their functional properties. As natural additives, EPS serve as effective thickeners, stabilizers, and emulsifiers in beverages, dairy products, and jellies, partially replacing synthetic additives while delivering prebiotic benefits that stimulate beneficial gut bacteria (Li et al. 2024; Xiao and Zheng 2016). Moreover, their demonstrated bioactive effects including antioxidant, antimicrobial, antiviral, and immunomodulatory activities underlie significant potential for developing functional foods and supplements that target chronic conditions such as asthma and diabetes while enhancing immunity (Capek et al. 2020; Li et al. 2024).

*Porphyridium* spp., a unicellular red microalga (Rhodophyta, Porphyridiaceae), is rich in phycobiliproteins and sulfated polysaccharides, and finds extensive applications in food and skincare (Li et al. 2019). Our research group previously established high-density cultivation techniques for the red species *P. purpureum* (Li et al. 2021), achieving selective accumulation of phycoerythrin and polysaccharides through carbon-to-nitrogen ratio regulation (Li et al. 2020), while also investigating the impact of different light spectra on phycobiliprotein and polysaccharide content in two *Porphyridium* species (Ji et al. 2024). However, the influence of photoperiod on *Porphyridium* growth and metabolism remains underexplored, and current research primarily focuses on the red species, whereas understanding of the similarly phycobiliprotein- and polysaccharide-rich green species (*Porphyridium aerugineum*) remains limited. Therefore, this study primarily examines the effects of different photoperiods on the growth, phycobiliprotein production, and polysaccharide production of these two chromatically distinct *Porphyridium* species, aiming to provide theoretical value for developing *Porphyridium* as a future food.

## Materials and methods

### Cultivation of *porphyridium* species

*P. purpureum* (FACHB-806) and *P. aerugineum* (FACHB-744) were obtained from the Freshwater Algae Culture Collection at the Institute of Hydrobiology in Wuhan, China. Cultivation was carried out at 25 °C with agitation at 140 rpm under continuous white light at an intensity of 100 μmol/m^2^/s, which was predetermined as optimal for growth in preliminary experiments (data not shown). *P. purpureum* and *P. aerugineum* were cultivated in their respective optimal growth media, ASW and KOCK.

To investigate the effects of different photoperiods, preserved *Porphyridium* stock cultures were first activated for 6 days in a larger air-lifted photobioreactor (500/800 mL). Following activation, the cultures were inoculated into smaller air-lifted photobioreactors (300/500 mL) at an initial OD_750_ of 0.50 for the main 8-day cultivation experiment. The smaller system was chosen for this phase to enable a faster response and clearer observation of growth differences within the shorter experimental timeframe. The cultures were subjected to three photoperiod regimes: long photoperiod (24/0 h light/dark), moderate photoperiod (12/12 h light/dark), and short photoperiod (6/18 h light/dark), all at a light intensity of 100 μmol/m^2^/s.

For the time-course cultivation under short and long photoperiods, which extended over 20 days, the cultures were instead scaled up to the larger photobioreactors (500/800 mL). This was necessary to accommodate the more frequent sampling (every 4 days) for physiological and biochemical analysis, as the larger volume minimized the potential impact of sampling and water evaporation on the culture stability. To compensate for visible water evaporation due to aeration, the culture volume was maintained by adding sterile deionized water as make-up water to the original calibration mark prior to each sampling or analysis (Ji et al. 2024).

### Growth and chlorophyll* a* content determination

The cell density of both *Porphyridium* species was characterized by OD_750_ with UV–visible spectrophotometer. High cell densities need to be diluted with medium before measurement. For biomass calculation, cells were centrifuged at 10,000 rpm for 2 min, washed with 20 mM Phosphate-buffered saline (PBS) (pH = 6.8) and resuspended to OD_750_ = 1.00, then 5 mL of the resuspension was filtered into a pre-dried and weighed JINJING nitrocellulose membrane filter (0.45 μM) and dried in a 55 °C oven for 48 h. The dry weight is obtained by subtracting the weight of the filter from the total weight of the filter loaded with algal cells after drying and multiplying by the dilution factor (Ji et al. 2024).

For the determination chlorophyll *a* concentration, 2 mL of algal cells (OD_750_ = 1.00) were centrifuged, washed with 20 mM phosphate-buffered saline (PBS, pH = 6.8), and then soaked in 90% acetone at 4 °C for 24 h in the dark. The supernatant was subsequently collected by centrifugation (4 °C, 5000 rpm for 10 min), and absorbance was measured at 665 nm using a spectrophotometer. Chlorophyll *a* concentration was further calculated according to our previous study (Ji et al. 2021).

### Structural and component characterization of phycobiliproteins

The algal cells were collected by centrifugation at 10,000 rpm for 5 min, washed with 20 mM PBS (pH = 6.8) and resuspended to OD_750_ = 1.00, and 10 mL of the resuspension was subjected to ultrasonication with parameters of 150 W, 18 min, and an on/off of 7 s/5 s. The supernatant was collected by centrifugation at 10,000 rpm for 10 min after ultrasonication, and 1 mL of the crude extract was scanned by a UV–visible spectrophotometer at a wavelength range of 200–800 nm, and another 1 mL of the crude extract was used to determine the absorbance at 565 nm, 620 nm, and 650 nm. The concentration of each phycobiliprotein was calculated according to the formula (Marsac and Houmard 1988). The content or concentration of total phycobiliprotein refers to the sum of the content or concentration of phycoerythrin, phycocyanin and allophycocyanin. It should be noted that the algae cells were resuspended to OD_750_ = 1 for the determination of various parameters because we previously found that the cell mass of *Porphyridium* significantly affects the efficiency of cell disruption. The current parameters can ensure the full extraction of bioactive substances without further damage (Ji et al. 2022).

### Quantification of exopolysaccharides, total soluble proteins, carbohydrates and neutral lipids

The crude extracts mentioned earlier were used for the determination of total soluble protein and carbohydrate content, which were determined by the Coomassie Brilliant Blue G-250 method (Grintzalis et al. 2015) and the phenol–sulfuric acid method (Dubois et al. 1956), respectively. Briefly, 100 μL of crude extract was taken and 1 mL of staining solution was added to it, which was left at room temperature for 5 min, and the absorbance at 595 nm was measured, and the protein content was calculated from the standard curve using 20 mM PBS (pH = 6.8) as the control.

For carbohydrate content, 1 mL of 20 mM PBS (pH = 6.8) was added to 1 mL of crude extract, followed by 1 mL of freshly prepared 6% phenol and 5 mL of concentrated sulfuric acid. The mixture was left at room temperature for 10 min, then 45 °C water bath for 30 min, and 1 mL was taken after cooling to determine the absorbance at 490 nm. The carbohydrate content was calculated from the standard curve using 20 mM PBS (pH = 6.8) as control. The supernatant of algal cells collected by centrifugation at 10,000 rpm for 5 min was used for the determination of crude exopolysaccharides in the same way as for carbohydrate content.

The algal cell resuspension (OD_750_ = 1.00) was used for the determination of neutral lipid content according to the Nile red method (Chen et al. 2009). Briefly, 1 mL of the resuspension was added with 10 μL of Nile Red dye (0.1 mg/mL), and after 30 min at 37 °C, 200 μL of the mixture was taken in a 96-well plate and read with a multi-mode microplate reader (Ex/Em = 530 nm/592 nm).

### Statistical analysis

Each experiment had three replicates and the data were expressed as the mean and standard deviation (SD). Statistical differences were determined by Student's t-test using GraphPad Prim statistic software, V10.2.3 (GraphPad Software, San Diego, California, USA). Data were considered significant when at least *P* was < 0.05 (* refer to *P* value < 0.05, ** refer to *P* value < 0.01, *** refer to *P* value < 0.001, and **** refer to *P* value < 0.0001).

## Results and discussion

### Comparative characterization of phycobiliprotein and chlorophyll* a* content in two *Porphyridium* species

**Fig. 1:**
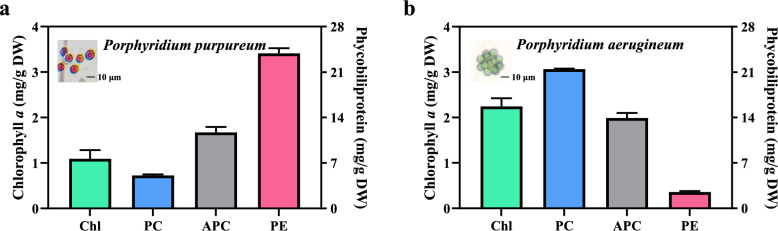
Characterization of light-harvesting antennae composition in two *Porphyridium* species under flask culture conditions. Chlorophyll *a* and phycobiliprotein content in *P. purpureum* (**a**) and *P. aerugineum* (**b**). All data were determined during the mid-logarithmic growth phase. The embedded image shows the morphology and color of *Porphyridium* under a microscope. Chl, PE, PC, and APC refer to chlorophyll *a*, phycoerythrin, phycocyanin, and allophycocyanin, respectively. Data are expressed as mean ± SD (n = 3)

Significant differences in phycobiliprotein and chlorophyll *a* content were observed between the two *Porphyridium* species. In *P. purpureum*, the contents of phycocyanin, allophycocyanin, and phycoerythrin were 5.08 ± 0.15 mg/g DW, 11.71 ± 0.84 mg/g DW, and 23.89 ± 0.79 mg/g DW, respectively. Phycoerythrin was the predominant phycobiliprotein in *P. purpureum*, comprising 58.74 ± 0.62% of the total, contributing to its bright red color (Fig. 1a). In contrast, *P. aerugineum* exhibited phycocyanin, allophycocyanin, and phycoerythrin contents of 21.46 ± 0.18 mg/g DW, 13.93 ± 0.79 mg/g DW, and 2.52 ± 0.14 mg/g DW, respectively. Phycocyanin served as the core phycobiliprotein in *P. aerugineum*, accounting for 56.62 ± 0.88% of the total (Fig. 1b). Additionally, the chlorophyll *a* content in *P. purpureum* (1.09 ± 0.19 mg/g DW) was lower than that of *P. aerugineum* (2.24 ± 0.18 mg/g DW). Combined with its high PC content, the higher chlorophyll *a* level imparts a vibrant green color to *P. aerugineum*.

Our results highlight distinct light-harvesting strategies: *P. purpureum* is rich in phycoerythrin, while *P. aerugineum* possesses higher levels of phycocyanin and chlorophyll *a* (Fig. 1). Notably, the phycoerythrin content in *P. purpureum* significantly exceeds that reported for red macroalgae and surpasses several other phycoerythrin-rich microalgae, such as the endophytic filamentous red alga *Colaconema* sp. (5.4–6.5 mg/g DW) (Lee et al. 2021) and the thermophilic cyanobacterium *Leptothermofonsia sichuanensis* (2.13–21.92 mg/g DW) (Yao et al. 2024). Although *P. aerugineum* exhibited a substantial phycocyanin content, significantly higher than *P. purpureum*, it remains lower than values reported for some cyanobacteria like *Spirulina* sp. (17.5% w/w), *Phormidium* sp. (4.1% w/w), and *Lyngbya* sp. (3.9% w/w) (Patel et al. 2005). It is important to note that phycobiliprotein content in microalgae can vary considerably depending on cultivation duration and environmental conditions (Hsieh-Lo et al. 2019; Klepacz-Smolka et al. 2020). Optimization of these factors suggests the phycocyanin content in *P. aerugineum* has significant potential for enhancement. Furthermore, *P. aerugineum* benefits from a simpler unicellular structure compared to the multicellular spiral or filamentous forms of *Spirulina* sp., *Phormidium* sp., and *Lyngbya* sp., potentially facilitating easier extraction. The combination of high phycobiliprotein content and relatively simple cellular morphology in both *Porphyridium* species positions them as highly promising candidates for efficient phycobiliprotein production.

### Effect of photoperiod on the growth of *P. purpureum* and *P. aerugineum*

**Fig. 2:**
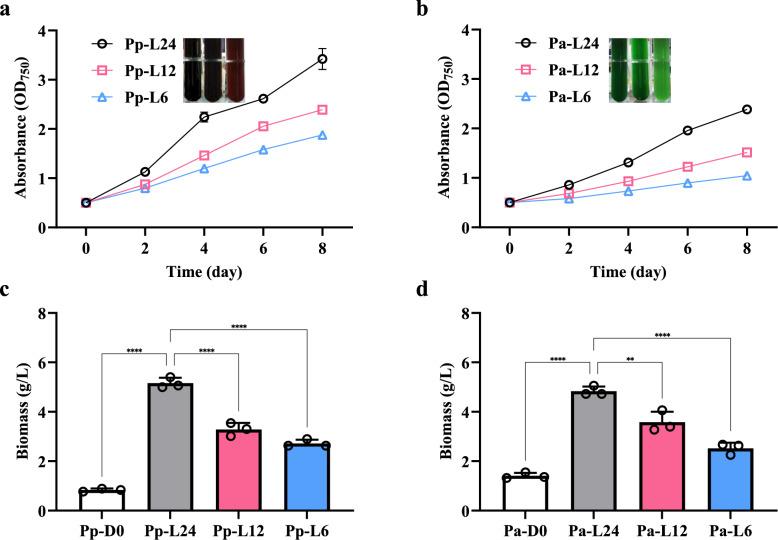
Growth curves and dry weights of two *Porphyridium* species cultured under different photoperiods. Growth curves of *P. purpureum* (**a**) and *P. aerugineum* (**b**). Dry weights of *P. purpureum* (**c**) and *P. aerugineum* (**d**) at the time of inoculation and on day 8. The embedded image illustrates the cultivation of *Porphyridium* on the 8th day in a column photobioreactor (300/500 mL). Pp-D0/Pa-D0, Pp-L24/Pa-L24, Pp-L12/Pa-L12, and Pp-L6/Pa-L6 refer to *P. purpureum/P. aerugineum* at the time of inoculation and cultured under long, moderate, and short photoperiods, respectively. Data are expressed as mean ± SD (n = 3)

Given the distinct light-harvesting antenna compositions of *P. purpureum* and *P. aerugineum*, we investigated whether they exhibit similar photoperiod responses by measuring growth curves and biomass accumulation under three photoperiod regimes. The results revealed that extended light duration enhanced growth rates and biomass accumulation in both species: *P. purpureum* under long photoperiods reached a biomass of 5.16 ± 0.22 g/L (dry weight) on day 8, significantly exceeding levels under moderate (3.29 ± 0.26 g/L) and short (2.72 ± 0.15 g/L) photoperiods, while *P. aerugineum* showed a parallel trend with biomass of 4.83 ± 0.19 g/L (long), 3.58 ± 0.42 g/L (moderate), and 2.52 ± 0.23 g/L (short) (Fig. 2).

**Fig. 3:**
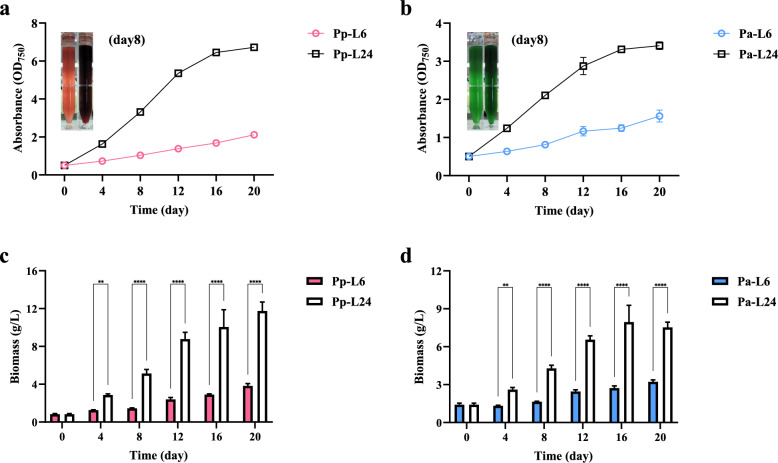
Growth curves and time-course changes in dry weight of two *Porphyridium* species cultured under short and long photoperiods. Growth curves of *P. purpureum* (**a**) and *P. aerugineum* (**b**). Time-course changes in dry weight of *P. purpureum* (**c**) and *P. aerugineum* (**d**). The embedded image illustrates the cultivation of *Porphyridium* on the 8th day in a column photobioreactor (500/800 mL). Pp-L6/Pa-L6 and Pp-L24/Pa-L24 and refer to *P. purpureum/P. aerugineum* cultured under short and long photoperiods, respectively. Data are expressed as mean ± SD (n = 3)

To further investigate the dynamics of phycobiliproteins under different photoperiods, extended cultivation was conducted in both long and short photoperiod conditions. Consistent with prior findings, *P. purpureum* and *P. aerugineum* reached the stationary phase faster under long photoperiods, while exhibiting prolonged lag phases under short photoperiods. Specifically, *P. purpureum* cultured under long photoperiods reached a maximum biomass of 11.75 ± 0.94 g/L on day 20, which was significantly higher than the 3.82 ± 0.26 g/L observed under short photoperiods. In contrast, *P. aerugineum* achieved its peak biomass values of 7.95 ± 1.32 g/L on day 16 (under long photoperiods) and 3.22 ± 0.15 g/L on day 20 (under short photoperiods), respectively (Fig. 3).

While the two *Porphyridium* species exhibit distinct phycobiliprotein compositions, their growth responds similarly to photoperiod variations, consistent with studies in *Desmodesmus* sp. CHX1 where extended photoperiods enhanced growth (Tian et al. 2024). However, broader studies demonstrate that microalgal growth under varying photoperiods typically increases initially before declining with prolonged light exposure, as seen in algae *Ankistrodesmus falcatus* and *Fragilariopsis cylindrus* peaking under 16L:8D cycles (George et al. 2014; Guérin et al. 2024), while microalgae *Platymonas helgolandica* thrives optimally under 15L:9D cycles, indicating species-specific photoperiod preferences (Chu et al. 2023). Crucially, these variations arise not only from genetic differences but also from interactions with light intensity and inoculum density; for example, Chen et al. (2023) observed photoinhibition in four *Chlorella* species under 20L:4D cycles at high light intensity (8000 lx) and low initial density (2 × 10^5^ cells/mL), leading to lower biomass under these conditions compared to 16L:8D cycles. In contrast, our study employed higher cell densities (approximately 1 × 10^6^ cells/mL) and lower light intensities (approximately 6000 lx), conditions under which neither *Porphyridium* species experienced photoinhibition despite differing phycobiliprotein content, thereby explaining their analogous photoperiod responses.

### Time-course changes in phycobiliproteins and chlorophyll* a* content

**Fig. 4:**
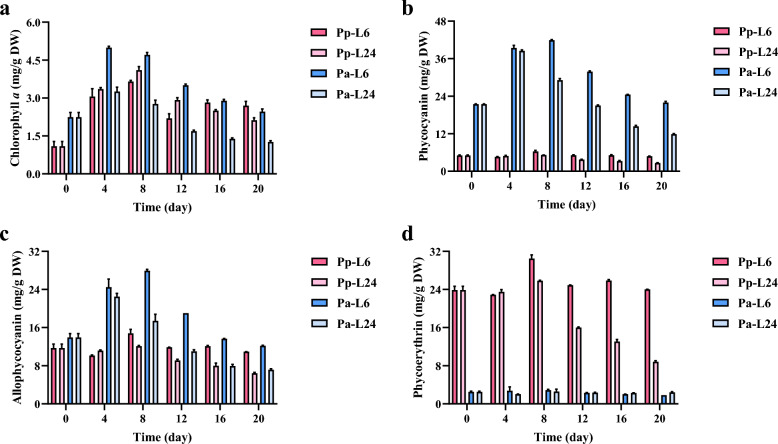
Time-course changes in the contents of light-harvesting antennae in *P. purpureum* and *P. aerugineum* cultured under short and long photoperiods. Changes in the contents of phycoerythrin (**a**), phycocyanin (**b**), allophycocyanin (**c**), and chlorophyll *a* (**d**). Pp-L6/Pa-L6 and Pp-L20/Pa-L20 refer to *P. purpureum/P. aerugineum* cultured under short and long photoperiods, respectively. Data are expressed as mean ± SD (n = 3). Means with the same letter are not significantly different

Both phycobiliproteins (PBP) and chlorophyll *a* serve as light-harvesting antennae in *Porphyridium* species, making their content inherently light-dependent. This light dependence is evident in both *P. purpureum* and *P. aerugineum*, which exhibit higher phycobiliprotein content under short photoperiods compared to long photoperiods (Fig. 4). This trend aligns with observations by Yeh et al. (2022) in the red alga *Colaconema formosanum*, where phycobiliprotein levels increased with decreasing light duration, peaking under complete darkness (e.g., phycoerythrin at 3.4 mg/g DW). Notably, *P. purpureum* achieves significantly higher phycoerythrin levels under short photoperiods, up to 30 mg/g DW, highlighting its strong potential for phycoerythrin production. A similar trend was observed in the cyanobacterium *Synechococcus* PCC 6715, where phycocyanin is the dominant phycobiliprotein (Klepacz-Smolka et al. 2020). Its phycocyanin and allophycocyanin content under a 16L:8D photoperiod was significantly higher than under continuous light. While *Synechococcus* PCC 6715 reached a maximum phycocyanin content of 31.5 mg/g DW, this value remains 17% lower than *P. aerugineum*'s maximum yield (38 mg/g DW) under equivalent short photoperiod conditions. The superior productivity demonstrates considerable promise for *P. aerugineum* in industrial phycocyanin production.

Furthermore, phycocyanin, the core phycobiliprotein in *P. aerugineum*, displayed biphasic cultivation dynamics featuring initial accumulation followed by progressive depletion (Fig. 4). Such kinetics indicate insufficient irradiance for this species, necessitating upregulated phycobiliprotein biosynthesis for light capture optimization. In *P. purpureum*, phycoerythrin (dominant phycobiliprotein) maintained stable early-phase content but declined progressively post-day 8. Consistent with adequate irradiance, this pattern reveals species-specific differences in photosynthetic photon flux density (PPFD) tolerance. Notably, *P. aerugineum* exhibited more pronounced chlorophyll *a* fluctuation paralleling phycocyanin/allophycocyanin dynamics, underscoring greater reliance on chlorophyll *a*-mediated harvesting under light limitation than observed in *P. purpureum*. These results indicate that the content of light-harvesting antennae, particularly phycobiliproteins, varies during cultivation in *Porphyridium*. Such variations are influenced not only by the photoperiod but also by the species' inherent preference for light intensity.

**Fig. 5:**
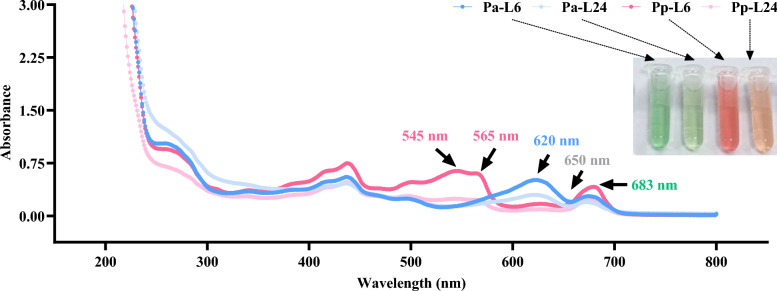
Absorption spectra of supernatants from ultrasonically disrupted *P. purpureum* and *P. aerugineum* cultured under short and long photoperiods on day 20. The embedded image displays the color of the supernatant from the crude extract. Pp-L6/Pa-L6 and Pp-L24/Pa-L24 and refer to *P. purpureum/P. aerugineum* cultured under short and long photoperiods, respectively

The absorption peaks at 545 nm and 565 nm are characteristic of phycoerythrin, while those at 620 nm, 650 nm, and 683 nm correspond to phycocyanin, allophycocyanin and chlorophyll *a*, respectively (Ji et al. 2023; Pekárková et al. 1989). Differences in the content of the light-harvesting antennas of *P. purpureum* and *P. aerugineum* cultured under different photoperiods can be observed from the changes in the heights of these characteristic absorption peaks. For *P. purpureum* cultured under short photoperiods, all absorption peaks were higher compared to those under long photoperiods, with more pronounced changes noted for phycoerythrin and chlorophyll *a*. In contrast, *P. aerugineum* displayed higher characteristic absorption peaks for phycocyanin and chlorophyll *a*. This indicates that the structure of the light-harvesting antennas is indeed influenced by the photoperiod and is more closely related to the core phycobiliproteins and chlorophyll *a*. Additionally, structural changes in the light-harvesting antennas are evident from the color of the crude extracts, with *P. purpureum* exhibited a brighter red and *P. aerugineum* a more vibrant emerald green under short photoperiod, respectively (Fig. 5).

Photoacclimation in microalgae refers to the process by which they regulate their metabolism through adjusting the content of light-harvesting pigment-proteins in response to varying light environments (Bennett and Bogorad 1973; Grossman et al. 1993). The underlying principle is that under light-limiting conditions, cells upregulate the synthesis of light-harvesting antennae to enhance light capture capacity; whereas under sufficient/excess light conditions, they downregulate light-harvesting protein content to prevent damage from reactive oxygen species (ROS) generated under excessive light (Fu and Wang 2023; Voerman et al. 2022). Previous research has demonstrated that low light intensity stimulates phycoerythrin synthesis in *P. purpureum* (Ji et al. 2022). Blue light exposure, compared to white light, also enhances phycobiliprotein production in both *P. purpureum* and *P. aerugineum* (Ji et al. 2024). Collectively, these observations indicate that short photoperiods may function as a form of photosynthetically active radiation (PAR) limitation. This photoperiod-induced light deficiency similarly elevates phycobiliprotein content in *Porphyridium* species to augment light harvesting efficiency during restricted illumination intervals.

### Time-course changes in exopolysaccharide content with co-quantified biomolecules

**Fig. 6:**
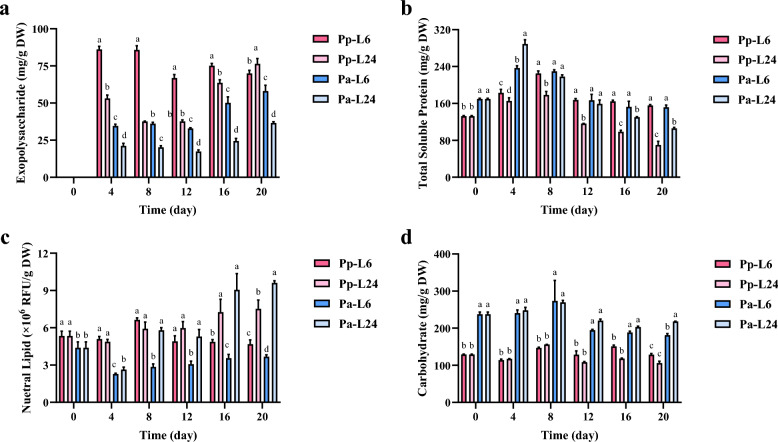
Time-course changes in the contents of biological macromolecules of *P. purpureum* and *P. aerugineum* cultured under short and long photoperiods. Changes in the contents of exopolysaccharide (**a**), total soluble protein (**b**), carbohydrate (**c**) and neutral lipid (**d**). Pp-L6/Pa-L6 and Pp-L24/Pa-L24 and refer to *P. purpureum/P. aerugineum* cultured under short and long photoperiods, respectively. Data are expressed as mean ± SD (n = 3). Means with the same letter are not significantly different

*Porphyridium* typically synthesizes and secretes substantial exopolysaccharides under stress conditions or upon entering the stationary phase (Ji et al. 2021; Li et al. 2023). In *P. purpureum*, the relatively higher exopolysaccharide content observed during early cultivation under short photoperiods results from two concurrent factors: enhanced EPS secretion as cells acclimate to environmental conditions, paired with slower cell division rates. This dual effect elevates initial exopolysaccharide content (mg/g DW). Conversely, cultures under long photoperiods progress more rapidly through the growth cycle, reaching the exponential phase sooner. Consequently, these cultures exhibit lower initial exopolysaccharide accumulation. However, as long-photoperiod cells subsequently enter the stationary phase earlier, reduced division rates combined with sustained exopolysaccharide secretion ultimately enable these cultures to surpass the exopolysaccharide content of short-photoperiod cultures. Parallel observations in *P. aerugineum* further validate this photoperiod-dependent pattern: Under short photoperiods, its cultures exhibited the lowest growth rate, maintaining an extended light-acclimation phase even after 20 days of cultivation. This prolonged adaptation period sustained elevated exopolysaccharide levels throughout. In contrast, long-photoperiod cultures underwent characteristic exopolysaccharide dynamics, marked by an initial decline followed by a subsequent increase, reaching stationary phase by day 20 (Fig. 6a). Projected accumulation trajectories suggest continued exopolysaccharide increase in long-photoperiod systems beyond this point, whereas short-photoperiod cultures may experience a subsequent decline due to accelerated cell division upon entering the exponential growth phase.

Total soluble protein in both *Porphyridium* species displayed characteristic rise-fall dynamics during cultivation. Significantly higher protein levels were maintained under short photoperiods compared to long-photoperiod regimes, attributable to delayed stationary-phase transition under reduced illumination. This photoperiodic effect was especially evident in *P. purpureum*, where long-photoperiod cultures exhibited a sharp decline in total soluble protein content to 70.13 ± 7.96 mg/g DW by day 20, which was merely 45.05% of levels under short photoperiods (Fig. 6b). Conversely, both species accumulated higher neutral lipid content under long photoperiods. This trend was particularly pronounced in *P. aerugineum*, which exhibited notably rapid neutral lipid accumulation during late cultivation, reaching 9.61 ± 0.18 RFU/g DW by day 20 (Fig. 6c). In contrast to total soluble proteins and neutral lipids, carbohydrate content in both algae showed minimal photoperiodic influence, remaining relatively stable throughout the cultivation period (Fig. 6d). Collectively, the rapid protein decline coupled with concurrent lipid accumulation under long photoperiods suggests that accelerated entry into stationary phase during extended light exposure triggers metabolic reprogramming, redirecting carbon flux from proteins toward energy storage lipids. This metabolic shift aligns with findings by He et al. (2015), who observed a similar initial increase followed by decrease in protein content over culture time in *Chlorella* sp. and *Monoraphidium* sp., indicating a conserved strategy where protein degradation upon stationary phase entry provides carbon precursors for lipid biosynthesis.

### Time-course cultivation reveals divergent production peaks for phycobiliprotein and exopolysaccharide

Phycoerythrin is the dominant phycobiliprotein in *P. purpureum*, while phycocyanin is predominant in *P. aerugineum*; both pigments are widely used in the food industry. Therefore, we determined time-course changes in phycoerythrin yield in *P. purpureum* and phycocyanin yield in *P. aerugineum*. The results showed that although phycobiliprotein content was higher under short photoperiods, phycobiliprotein yield was ultimately greater under long photoperiods due to significantly faster biomass accumulation. Furthermore, phycobiliprotein yield initially increased before decreasing over the cultivation period, peaking on day 12: phycoerythrin yield in *P. purpureum* reached 140.6 ± 0.89 mg/L, while phycocyanin content in *P. aerugineum* reached 137.7 ± 1.2 mg/L (Tables 1 and 2). Analysis indicates that the yield increase was primarily driven by rapid biomass accumulation, whereas the later decline was mainly attributed to a significant decrease in phycobiliprotein content (Figs. 3 and 4). Notably, Xu et al. (2019) proposed an induction strategy involving reduced temperature and light intensity in later cultivation stages to slow the decline in phycoerythrin content, thereby increasing phycoerythrin yield in *P. purpureum* to 229 mg/L by day 12. This suggests that modifying the photoperiod through reduced light duration in later stages may further enhance phycoerythrin yield.

**Table 1:** Phycoerythrin production (mg/L) in *P. purpureum* under contrasting photoperiods

Species	Phycoerythrin (mg/L)					
	Day 0	Day 4	Day 8	Day 12	Day 16	Day 20
Pp-L6	19.9 ± 0.7	28.8 ± 0.1	44.3 ± 1.1	59.5 ± 0.1	75.1 ± 0.6	91.8 ± 0.0
Pp-L24	19.9 ± 0.7	67.2 ± 1.4	132.6 ± 0.6	140.6 ± 0.9	131.6 ± 4.4	104.0 ± 2.3

Pp-L6 and Pp-L24 refer to *P. purpureum* cultured under short and long photoperiods, respectively. Data are expressed as mean ± SD (n = 3)

**Table 2:** Phycocyanin production (mg/L) in *P. aerugineum* under contrasting photoperiods

Species	Phycocyanin (mg/L)					
	Day 0	Day 4	Day 8	Day 12	Day 16	Day 20
Pa-L6	30.3 ± 0.1	52.3 ± 1.1	69.1 ± 0.3	78.2 ± 0.7	67.0 ± 0.1	70.9 ± 1.1
Pa-L24	30.3 ± 0.1	100.3 ± 0.9	124.7 ± 2.0	137.7 ± 1.2	114.2 ± 2.2	89.1 ± 1.7

Pa-L6 and Pa-L24 refer to *P. aerugineum* cultured under short and long photoperiods, respectively. Data are expressed as mean ± SD (n = 3)

While exopolysaccharide content is generally higher under short photoperiods, exopolysaccharide yield consistently remains higher under long photoperiods. This outcome results from accelerated biomass accumulation during early cultivation stages coupled with enhanced exopolysaccharide production capacity in later phases. Compared to *P. aerugineum*, *P. purpureum* demonstrates superior exopolysaccharide productivity, achieving 898.7 ± 41.0 mg/L by day 20 under long photoperiods. This output exceeds *P. aerugineum*'s yield of 275.7 ± 6.2 mg/L under identical conditions by over threefold, matching *P. purpureum*'s short photoperiod yield at the same timepoint (Table 3). Despite this disparity, *P. aerugineum* remains a promising polysaccharide source, as our preliminary data indicates its exopolysaccharides contain elevated glucose and glucuronic acid content relative to *P. purpureum*'s, correlating with significantly stronger antioxidant activity (manuscript in preparation).

**Table 3:** Exopolysaccharide production (mg/L) in *P. purpureum* and *P. aerugineum* under contrasting photoperiods

Species	Exopolysaccharide (mg/L)					
	Day 0	Day 4	Day 8	Day 12	Day 16	Day 20
Pp-L6	0.0 ± 0.0	108.5 ± 2.6	124.6 ± 4.1	159.8 ± 5.6	218.0 ± 4.7	267.2 ± 7.7
Pp-L24	0.0 ± 0.0	151.8 ± 6.5	193.3 ± 1.0	330.9 ± 8.2	640.8 ± 20.9	898.7 ± 41.0
Pa-L6	0.0 ± 0.0	45.9 ± 1.7	59.5 ± 1.5	80.7 ± 1.2	136.8 ± 10.8	187.4 ± 12.4
Pa-L24	0.0 ± 0.0	55.1 ± 4.6	86.6 ± 4.6	113.8 ± 7.2	194.5 ± 13.6	275.7 ± 6.2

Pp-L6/Pa-L6 and Pp-L20/Pa-L20 refer to *P. purpureum/P. aerugineum* cultured under short and long photoperiods, respectively. Data are expressed as mean ± SD (n = 3)

## Conclusion

This study investigated time-course changes in the physiological and biochemical characteristics of two *Porphyridium* species under different photoperiods. Both species upregulated phycobiliprotein and chlorophyll *a* content to enhance light harvesting under short photoperiods, increasing maximum phycoerythrin levels in *P. purpureum* to 30.48 ± 0.79 mg/g DW and phycocyanin in *P. aerugineum* to 41.95 ± 0.16 mg/g DW. Conversely, rapid biomass accumulation under long photoperiods elevated phycobiliprotein production, with peak phycoerythrin titer reaching 140.61 ± 0.88 mg/L (*P. purpureum*) and phycocyanin 137.72 ± 1.20 mg/L (*P. aerugineum*). Both species serve as viable exopolysaccharide sources, though *P. purpureum* demonstrated superior productivity, yielding 898.7 ± 41.0 mg/L by day 20 under long photoperiods. These findings provide strategic insights for optimizing commercial production of phycobiliproteins and exopolysaccharides in *Porphyridium*.

## Data Availability

Data will be made available on request.
